# Supplementary material for: Life-history adaptation under climate warming magnifies the agricultural footprint of a cosmopolitan insect pest
Source: Nat Commun. 2025 Jan 18;16:827. doi: 10.1038/s41467-025-56177-2 (PMC11743133; doi:10.1038/s41467-025-56177-2)
Supplement: Supplementary file 1 — Supplementary Information [file 41467_2025_56177_MOESM1_ESM.pdf]

Life history adaptation under climate warming magnifies the agricultural footprint of a cosmopolitan insect pest

*Estelle Burc[1,2†], Camille Girard-Tercieux[1,3,4†], Moa Metz[1,5], Elise Cazaux[1,3], Julian Baur[1], Mareike Koppik[1,6], Alexandre Rêgo[1], Alex Hart[1] & David Berger[1]**

1. Department of Ecology and Genetics, Program of Animal Ecology. Uppsala University, Norbyvägen 18D, 75236, Uppsala, Sweden.
2. Agronomy Institute Rennes-Angers (IARA), Graduate school of agronomy, 35000 Rennes, France
3. Université de Toulouse, Toulouse INP-ENSAT, 31326 Castanet-Tolosan, France
4. Université de Lorraine, AgroParisTech, INRAE, UMR Silva, 54000 Nancy, France
5. Department of Biology, Faculty of Natural Sciences, Norwegian University of Science and Technology, 7491 Trondheim, Norway
6. Department of Zoology, Animal Ecology, Martin-Luther University Halle-Wittenberg, Halle (Saale), Germany.

†Both authors contributed equally and are presented in alphabetical order.

*Corresponding author.

david.berger@ebc.uu.se

Index:

<u>Model predictions:</u>

Supplementary Note 1: *Predictions for how thermal adaptation via increased energy acquisition affects the agricultural footprint of insect pests.*
Supplementary Code 1: R-code underlying model predictions uploaded separately.
Supplementary References.

<u>Gene expression:</u>
Supplementary Data 1: Gene annotations for all heat stress and reproduction genes (see separate file).

Supplementary Data 2: Gene ontology for all heat stress and reproduction genes (see separate file).

Supplementary Table 1: Additional analyses of gene expression data

Supplementary Figure 1: Allocation trade-off between reproduction and heat stress response in experimental evolution lines based on expression of overlapping genes.

Supplementary Figure 2: Allocation trade-off between reproduction and heat stress response in experimental evolution lines based on all significant genes.

<u>Life history Evolution:</u>
Supplementary Table 2: *Univariate models of thermal plasticity in life history traits*

Supplementary Table 3: *Univariate models of thermal adaptation in life history traits*

Supplementary Figure 3: *Estimates of laboratory fitness in experimental evolution lines*

<u>Host Consumption and agricultural footprint:</u>
Supplementary Table 4: *Estimates of host consumption in experimental evolution lines*

Supplementary Figure 4: Mean body mass of beetles emerging from the assays of host consumption.

<u>Selection on female body size:</u>
Supplementary Figure 5: Selection on female body mass at different temperatures

Supplementary Table 5: Selection on adult body mass

Supplementary Note 1:

Predictions of how thermal adaptation via increased resource acquisition affects the agricultural impact of insect pests based on protein biophysics.

Biological rates of ectotherms show an empirically well-described relationship with temperature that closely mirrors the thermodynamic performance of enzymes (Hochachka and Somero 2002; Angilletta 2009), because biological rates are governed at the biochemical level by the enzymatic reaction rate, k_{cat} (Evans and Polanyi 1935; Eyring 1935):

$$k_{\text{cat}} \propto A_B \, e^{-Ea/RT} \qquad \text{(Eq. S1)}$$

where E_a is the activation energy required for the enzymatic reaction to occur, R is the universal gas constant (0.002 kcal mol^{-1}), T is temperature in Kelvin and A_B is a species and rate specific constant for enzyme catalys (Clarke 2004; Dell et al. 2011). Equation 1 thus describes an increase in reaction rate with temperature (**Fig. 1A**, main text). The observed decline in biological rate at temperatures exceeding the organism's thermal optimum is attributed to a reduction in the proportion of functional enzyme due to reversable inactivation via protein unfolding (Bloom et al. 2005; DePristo et al. 2005; Bershtein et al. 2017; Echave and Wilke 2017; Agozzino and Dill 2018) (**Fig. 1A**). The proportion of folded protein ready to do work in a biological process follows a Boltzmann probability as a function of the Gibbs free energy of folding, ΔG, which is thus a measure of protein stability (DePristo et al. 2005):

$$\text{P}_{\text{fold}} = \prod_{i=1}^{n} \frac{1}{1+A_{D,i}\, e^{\Delta G_i(T)/RT}}. \qquad \text{(Eq. S2)}$$

Where constant A_D and stability, ΔG, is given for each of a set of *n* proteins that act in sequence and contribute multiplicatively to a biological rate (Chen and Shakhnovich 2010; Echave and Wilke 2017). At a benign temperature of 25˚C, most natural proteins occur in functional (properly folded) state and the mean value of the Gibbs energy is negative (mean ΔG ~ -7 kcal mol^{-1}; (DePristo et al. 2005; Chen and Shakhnovich 2009). However, ΔG is comprised of an enthalpy term (ΔH) and a temperature-dependent entropy term (ΔS) (Eyring and Polanyi 2013) that reduces to: $\Delta G(T) = \Delta H - T\Delta S$ over the ecologically relevant temperature range of most organisms (Chen and Shakhnovich 2010). Based on values from the literature (Chen and Shakhnovich 2010; Dill et al. 2011), ΔS ~ -0.25 to -0.50 kcal/mol K^{-1} for a protein of typical length. From equation S2 it is therefore clear that warm temperatures increase entropy making ΔG less negative, leading to a rapid non-linear reduction in the fraction of functional protein (**Fig. 1A**).

The reaction rate kinetics of equation S1 have been combined with the protein folding stability of equation S2 to describe temperature-dependent biological rates and even fitness in simple scenarios (Bershtein et al. 2017; Echave and Wilke 2017). We here place these biophysical predictions into a life history framework incorporating acquisition and allocation trade-offs (Jong and Noordwijk 1992) to predict the temperature dependence of population growth rate:

$$r(T) \propto (1-c)^M * M^b * k_{\text{cat}} * \text{P}_{\text{fold}} \qquad \text{(Eq. S3)}$$

where *M* is accumulated energy reserves, *c* is the survival cost of acquiring energy, and *b* is the allometric exponent for how growth and reproduction scale with *M*.

While there is variation in biological rates across organisms and for different traits (Clarke 2004; Angilletta 2009; Huey and Kingsolver 2011), inherent enzyme properties governing the temperature-dependence of protein stability and reaction rate have been shown to evolve slowly (Dill et al. 2011). Instead, thermal adaptation often proceeds via costly compensatory responses involving upregulation of molecular chaperones such as heat-shock proteins that aid protein folding at hot temperature (Feder et al. 2000), and enzyme catalysts that allow reactions to take place at cold temperature despite thermodynamic constraints (Hochachka and Somero 2002). Here we illustrate how such thermal compensation via increased energy acquisition can affect growth rates of ectotherm pests facing changing climates (see **Fig. 1C**, main text). Assume that the energy reserves, *M*, of an organism can be allocated with fraction *p* to produce molecules that increase enzyme reaction rate, and with fraction *q* to molecules that aid protein folding stability, leaving *M*(1-*p*-*q*) reserves for growth and reproduction. Inserting these expressions into equation S3 gives:

$$r'(T) = (1-c)^M * [M(1-p-q)]^b * pMA_B\, e^{-\frac{Ea}{RT}} * \prod_1^i \frac{1}{1+\frac{A_D}{qM}e^{\Delta G_i(T)/RT}} \qquad \text{(Eq. S4)}$$

We can find the optimal strategy of energy acquisition (increasing *M* at cost *c*) and allocation (of *M* to *p* and *q*) at different temperatures and for different costs of growth by numerically solving for the combination of *M*, *p* and *q* that maximize r'.

Eq. S4 is well-supported in its qualitative sense. Eqs. S1 and S2 have received strong empirical support from in vitro measures of enzymes (Dill et al. 2011; Echave and Wilke 2017; Agozzino and Dill 2018) and recapitulates the asymmetry of empirically estimated thermal reaction norms of quantitative traits (Angilletta 2009). Thermal compensation via molecular chaperones is also well-understood (Hochachka and Somero 2002) and has been shown to trade-off with growth and reproduction (Feder et al. 2000). However, the quantitative solution to the multidimensional trade-off described by eq. S4 depends on the mechanistic basis of thermal sensitivity down to its finer details and exactly how energy reserves invested in thermal compensation affect folding stability and reaction rate, relationships that are not empirically well-defined nor completely understood (e.g. Eqs. S1 and S2 describe phenomenological rather than mechanistic relationships). Thus, our aim is only to provide qualitative predictions. We do this by using eq. S4 to illustrate how thermal niches can evolve via compensatory energy acquisition and allocation under a relatively high (*c* = 0.9) or low (*c* = 0.8) cost of energy acquisition, when an organism is adapting to a cold (23°C) or hot (35°C) temperature (**Fig. 1C**, main text). For other parameters we first assumed values that correspond to those commonly observed in the literature: $\Delta G_{T=298K}$ = -7 kcal mol^{-1}; $\Delta S \sim -0.25$ kcal mol^{-1} K^{-1}; *n* = 100 proteins; *b* = 0.75, *Ea* = 20 kcal mol^{-1}(~ 0.7eV). A_B, and A_D were set to 1 for simplicity.

After finding the optimal strategy in the four scenarios, resulting thermal reaction norms for population growth rate were calculated and are depicted in **Fig. 1C** of the main text, along with the optimal energy acquisition strategy in each scenario. Cold-adapted populations outcompete hot-adapted population at cold temperature, but growth rates and the predicted

impact on agriculture are greater at warm temperatures and for the hot-adapted population due to reduced thermodynamic constraints on reaction rates (Gillooly et al. 2002; Brown et al. 2004). Lower costs of feeding favour increased energy acquisition and allocation to maintenance, which improves thermal performance and increases thermal niche breadth (**Fig. 1C,** main text). Increased feeding effort is particularly beneficial at hot temperature because there are increased demands on maintaining protein folding at these temperatures (**Fig. 1A,** main text). Thermal adaptation via increased energy acquisition mediates an even greater impact on the rate of host consumption (given by the product of r' and *M* – the latter assumed to be proportional to per capita host consumption) (**Fig. 1D,** main text**, Fig. C-F**).

To further illustrate how warming temperatures affect energy acquisition and the predicted impact on agriculture, we calculated the optimal life-history strategy (*M', p'* and *q'*) at each temperature under different assumptions of the thermal sensitivity of protein folding (Supplementary Note 1 Figure, below). We then calculated the associated population growth rate and rate of host consumption for each temperature and scenario. This confirmed that, if increased energy acquisition and allocation can compensate effects of temperature on molecular failure rates, greater thermal sensitivity favours increased foraging effort **(Fig. A, B**). Increased thermal sensitivity implies low fitness at high temperature per definition, so population growth rates remain low in this scenario (**Fig. C, D**) as thermal compensation via increased growth effort also comes at a cost (*c*). However, if costs of feeding are low, increased energy acquisition is more favourable, which increases population growth rate, and even more so, agricultural impact (**Fig. E, F**). In fact, the strategy to increase *M* at stressful temperature causes populations experiencing temperatures slightly hotter than those that maximize population growth rate to have the greatest agricultural impact (compare **Fig. C** vs. **E** & **Fig. D** vs. **F**).

We note that protein folding can also be compromised by acute cold stress (Feder et al. 2000; Hochachka and Somero 2002). However, such cold temperatures are typically not experienced during the active seasons of tropical and sub-tropical insects (Deutsch et al. 2008; Johansson et al. 2020), as the case for *C. maculatus* (Baur et al. 2023), and were therefore not modelled. Likewise, stressful temperatures affect organisms in a multitude of ways. Thus, our particular example using protein folding is merely meant to illustrate a general principle and was chosen based on the simple fact that the modelled relationships are empirically well-supported in qualitative sense (see above). Other physiological phenomena that scale with temperature, such as metabolic expenditure (Gillooly et al. 2001) and the production of harmful reactive oxygen species (Dowling and Simmons 2009), are likely to simultaneously contribute to increasing molecular failure rates at stressful temperature.

Finally, similar to previous models (Bershtein et al. 2017; Echave and Wilke 2017), we here assumed that growth and reproduction of complex organisms are proportional to the rate at which they generate functional enzyme, which is clearly simplistic considering the ecological complexity affecting the link between rates of biochemical reactions and population growth rate. Yet, this simplicity allows forming general predictions and is supported qualitatively by the fact that a range of different biological rates scale predictably with temperature (Brown et al. 2004; Angilletta 2009). R code used to produce predictions is available as Supplementary Code 1.

Optimal energy acquisition and its agricultural impact

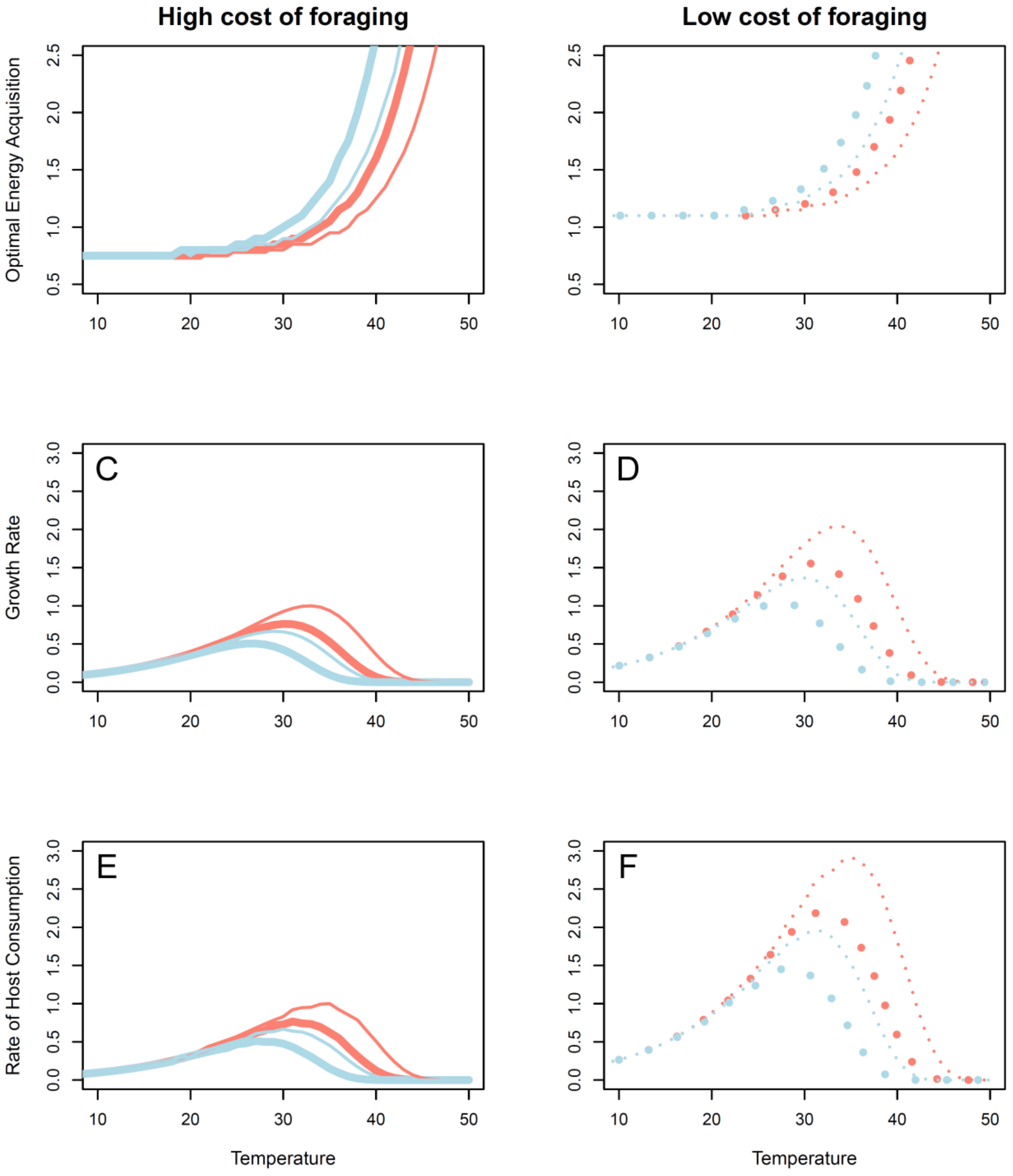

In **A)** and **B),** the optimal strategy of energy acquisition (*M'*) at each temperature in a scenario of high (c = 0.9; left panels) or low (c = 0.8; right panels) costs of foraging. 100 (thin lines) or 300 (thick lines) multiplicatively acting proteins were assumed to contribute to growth and/or reproduction. These proteins were either relatively stable ($\Delta G_{T=298K}$ = -7 kcal mol^{-1}; salmon) or less stable ($\Delta G_{T=298K}$ = -6 kcal mol^{-1}; light blue). More numerous, and less stable, proteins contribute to increased thermal sensitivity and benefit increases in *M'*. In **C)** and **D),** the resulting population growth rate, and in **E)** and **F)** the rate of host consumption, are shown at each temperature for each scenario. Note that these results represent optimal solutions to the multidimensional life-history trade-offs and could either stem from genetic adaptation or adaptive plasticity. Results in panels C-F are scaled by the maximum of the scenario depicted by thin and full lines in salmon colour ($\Delta G_{T=298K}$ = -7 kcal mol^{-1}; n = 100, c = 0.9).

Supplementary references:

Agozzino, L., and K. A. Dill. 2018. Protein evolution speed depends on its stability and abundance and on chaperone concentrations. PNAS 115:9092–9097.

Angilletta, M. J. 2009. Thermal Adaptation: A Theoretical and Empirical Synthesis. OUP Oxford.

Baur, J., M. Zwoinska, M. Koppik, R. R. Snook, and D. Berger. 2023. Heat stress reveals a fertility debt owing to postcopulatory sexual selection. Evolution Letters qrad007.

Bershtein, S., A. W. Serohijos, and E. I. Shakhnovich. 2017. Bridging the physical scales in evolutionary biology: from protein sequence space to fitness of organisms and populations. Curr. Opin. Struct. Biol. 42:31–40.

Bloom, J. D., J. J. Silberg, C. O. Wilke, D. A. Drummond, C. Adami, and F. H. Arnold. 2005. Thermodynamic prediction of protein neutrality. Proceedings of the National Academy of Sciences of the United States of America 102:606–611.

Brown, J. H., J. F. Gillooly, A. P. Allen, V. M. Savage, and G. B. West. 2004. Toward a Metabolic Theory of Ecology. Ecology 85:1771–1789.

Chen, P., and E. I. Shakhnovich. 2009. Lethal Mutagenesis in Viruses and Bacteria. Genetics 183:639–650.

Chen, P., and E. I. Shakhnovich. 2010. Thermal adaptation of viruses and bacteria. Biophysical journal 98:1109–1118.

Clarke, A. 2004. Is there a Universal Temperature Dependence of metabolism? Functional Ecology 18:252–256.

Dell, A. I., S. Pawar, and V. M. Savage. 2011. Systematic variation in the temperature dependence of physiological and ecological traits. Proceedings of the National Academy of Sciences 108:10591–10596.

DePristo, M. A., D. M. Weinreich, and D. L. Hartl. 2005. Missense meanderings in sequence space: a biophysical view of protein evolution. Nature Reviews Genetics 6:678–687.

Deutsch, C. A., J. J. Tewksbury, R. B. Huey, K. S. Sheldon, C. K. Ghalambor, D. C. Haak, and P. R. Martin. 2008. Impacts of climate warming on terrestrial ectotherms across latitude. PNAS 105:6668–6672.

Dill, K. A., K. Ghosh, and J. D. Schmit. 2011. Physical limits of cells and proteomes. PNAS 108:17876–17882.

Dowling, D. K., and L. W. Simmons. 2009. Reactive oxygen species as universal constraints in life-history evolution. Proceedings of the Royal Society B: Biological Sciences 276:1737–1745.

Echave, J., and C. O. Wilke. 2017. Biophysical models of protein evolution: understanding the patterns of evolutionary sequence divergence. Annual review of biophysics 46:85–103.

Evans, M. G., and M. Polanyi. 1935. Some applications of the transition state method to the calculation of reaction velocities, especially in solution. Trans. Faraday Soc. 31:875–894. The Royal Society of Chemistry.

Eyring, H. 1935. The Activated Complex in Chemical Reactions. J. Chem. Phys. 3:107–115. American Institute of Physics.

Eyring, H., and M. Polanyi. 2013. On Simple Gas Reactions. Zeitschrift für Physikalische Chemie 227:1221–1246. De Gruyter.

Feder, M. E., A. F. Bennett, and R. B. Huey. 2000. EVOLUTIONARY PHYSIOLOGY1. 29.

Gillooly, J. F., J. H. Brown, G. B. West, V. M. Savage, and E. L. Charnov. 2001. Effects of Size and Temperature on Metabolic Rate. Science 293:2248–2251.

Gillooly, J. F., E. L. Charnov, G. B. West, V. M. Savage, and J. H. Brown. 2002. Effects of size and temperature on developmental time. Nature 417:70–73.

Hochachka, P. W., and G. N. Somero. 2002. Biochemical Adaptation: Mechanism and Process in Physiological Evolution. Oxford University Press.

Huey, R. B., and J. G. Kingsolver. 2011. Variation in universal temperature dependence of biological rates. Proceedings of the National Academy of Sciences 108:10377–10378.

Johansson, F., G. Orizaola, and V. Nilsson-Örtman. 2020. Temperate insects with narrow seasonal activity periods can be as vulnerable to climate change as tropical insect species. Sci Rep 10:8822. Nature Publishing Group.

Jong, G. de, and A. J. van Noordwijk. 1992. Acquisition and Allocation of Resources: Genetic (CO) Variances, Selection, and Life Histories. The American Naturalist 139:749–770.

Supplementary Data 1: Gene annotations for all heat stress and reproduction genes (see separate file). P-values are based on hypergeometric tests for over-representation and corrected for multiple testing using the Benjamini-Hochberg method (false discovery rates).

Supplementary Data 2: Gene ontology for all heat stress and reproduction genes (see separate file). P-values are based on hypergeometric tests for over-representation and corrected for multiple testing using the Benjamini-Hochberg method (false discovery rates).

Supplementary Table 1: Gene expression analyses

Supplementary Table 1a: Nested ANOVA of allocation response based on the 134 antagonistic genes (corresponds to Figure 3D of main text. The model was fit using the "aov" function and was specified as:

```
Allocation ~ Evolution.regime*temperature*Origin + Error(Population)
```

Error: Population

	Df	Sum Sq	Mean Sq	F value	Pr(>F)	
Regime	2	1320027	660013	0.122	0.8867	
Temperature	2	140331406	70165703	12.972	0.0030	**
Origin	2	311216068	155608034	28.768	0.0002	***
Regime:Origin	4	238677450	59669362	11.031	0.0024	**
Residuals	8	43272241	5409030			

Error: Within

	Df	Sum Sq	Mean Sq	F value	Pr(>F)	
Temperature	2	4.3e+09	2.2e+09	262.982	1.63e-13	***
Regime:Temperature	4	1.7e+07	4.2e+06	0.514	0.7264	
Temeprature:Origin	4	9.5e+07	2.4e+07	2.867	0.0553	.
Regime:Temperature:Origin	8	1.1e+08	1.4e+07	1.738	0.1610	
Residuals	17	1.4e+08	8.3e+06			

Supplementary Table 1b: Nested ANOVA of allocation response based on all the 21 overlapping genes upregulated in response to reproduction. The model was fit using the "aov" function and was specified as:

```
Allocation ~ Evolution.regime*temperature*Origin + Error(Population)
```

Error: Population

	Df	Sum Sq	Mean Sq	F value	Pr(>F)	
Regime	2	251496	125748	11.964	0.00394	**
Temperature	2	23331	11666	1.110	0.37548	
Origin	2	56967	28483	2.710	0.12629	
Regime:Origin	4	115818	28954	2.755	0.10385	
Residuals	8	84084	10510			

Error: Within

	Df	Sum Sq	Mean Sq	F value	Pr(>F)	
Temperature	2	1117957	558978	72.545	4.74e-09	***
Regime:Temperature	4	33218	8304	1.078	0.3983	
Temperature:Origin	4	73571	18393	2.387	0.0918	.
Regime:Temperature:Origin	8	38745	4843	0.629	0.7433	
Residuals	17	130990	7705			

Supplementary Table 1c: Nested ANOVA of allocation response based on all the 113 overlapping genes upregulated in response to heat shock. The model was fit using the "aov" function and was specified as:

```
Allocation ~ Evolution.regime*temperature*Origin + Error(Population)
```

Error: Population

	Df	Sum Sq	Mean Sq	F value	Pr(>F)	
Regime	2	2697138	1348569	0.251	0.783913	
Temperature	2	137047700	68523850	12.755	0.003248	**
Origin	2	305102729	152551364	28.396	0.000232	***
Regime:Origin	4	235845545	58961386	10.975	0.002475	**
Residuals	8	42979006	5372376			

Error: Within

	Df	Sum Sq	Mean Sq	F value	Pr(>F)	
Temperature	2	4.21e+09	2.1e+09	258.02	1.91e-13	***
Regime:Temperature	4	1.63e+07	4.08e+06	0.500	0.736	
Temperature:Origin	4	9.14e+07	2.28e+07	2.800	0.059	.
Regime:Temperature:Origin	8	1.15e+08	1.44e+07	1.761	0.155	
Residuals	17	1.39e+08	8.16e+06			

Supplementary Table 1d: Nested ANOVA of allocation to reproduction based on all the 1269 differentially expressed genes in response to mating. The model was fit using the "aov" function and was specified as:

Allocation ~ Evolution.regime*Temperature*Origin + Error(Population)

Error: Population

	Df	Sum Sq	Mean Sq	F value	Pr(>F)	
Regime	2	1.4e+10	6.8e+09	3.82	0.0685	.
Temperature	2	9.1e+09	4.5e+09	2.52	0.142	
Origin	2	1.4e+10	6.8e+09	3.76	0.070	.
Regime:Origin	4	6.2e+09	1.5e+09	0.86	0.527	
Residuals	8	1.4e+10	1.8e+09			

Error: Within

	Df	Sum Sq	Mean Sq	F value	Pr(>F)	
Temperature	2	7.9e+10	3.6e+10	23.11	1.4e-05	***
Regime:Temperature	4	4.8e+09	1.2e+09	0.69	0.61	
Temepráture:Origin	4	4.2e+09	1.1e+09	0.62	0.65	
Regime:Temperature:Origin	8	7.8e+09	9.7e+08	0.57	0.79	
Residuals	17	2.9e+10	1.7e+09			

Supplementary Table 1e: Nested ANOVA of allocation to stress response based on all the 765 differentially expressed genes in response to heat shock. The model was fit using the "aov" function and was specified as:

Allocation ~ Evolution.regime*temperature*Origin + Error(Population)

Error: Population

	Df	Sum Sq	Mean Sq	F value	Pr(>F)	
Regime	2	5563026	2781513	1.31	0.32080	
Temperature	2	24185478	12092739	5.72	0.02872	*
Origin	2	93953143	46976571	22.21	0.00054	***
Regime:Origin	4	50331286	12582822	5.95	0.01601	*
Residuals	8	16921967	2115246			

Error: Within

	Df	Sum Sq	Mean Sq	F value	Pr(>F)	
Temperature	2	1.08e+09	5.42e+08	196.21	1.8e-12	***
Regime:Temperature	4	8.28e+06	2.07e+06	0.75	0.572	
Temepráture:Origin	4	3.24e+07	8.10e+06	2.94	0.052	.
Regime:Temperature:Origin	8	3.66e+07	4.58e+06	1.66	0.181	
Residuals	17	4.69e+07	2.76e+06			

Supplementary Figure 1: Allocation trade-off between reproduction and heat stress response in experimental evolution lines based on overlapping genes.

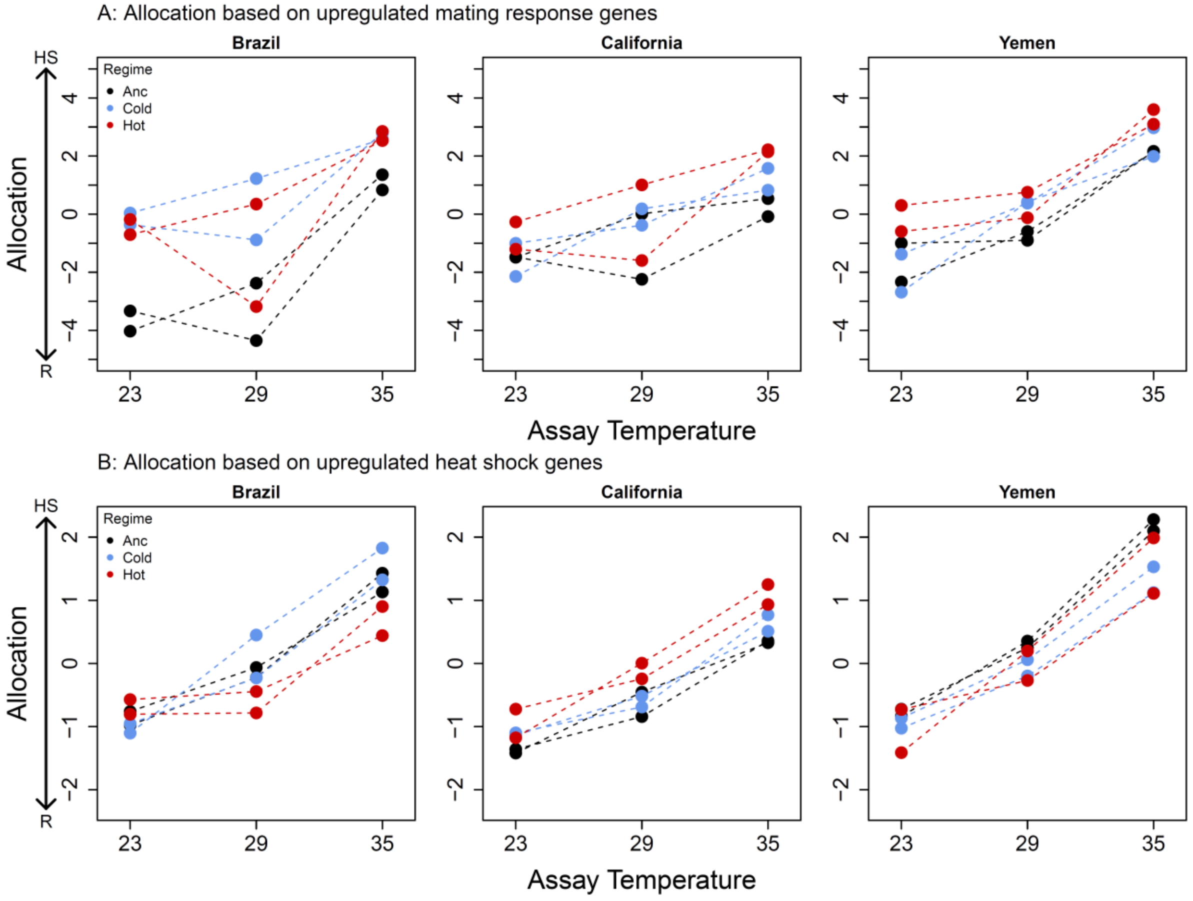

A: allocation based on expression of the 21 genes upregulated in response to mating in hot-adapted (red), cold-adapted (blue) and ancestors (black), reared at 23, 29, or 35°C. B: the same patterns for 113 genes upregulated in response to heat shock. Allocation towards stress response increased with assay temperature for both sets of genes. There was not general effect of evolution regime across the three backgrounds when analyzing the 113 genes upregulated in response to heat shock, while ancestors showed significantly higher expression of the 21 genes upregulated in response to mating. Each line's gene expression was based on transcriptomic data from a single RNA library consisting of 10 pooled female abdomens.

Supplementary Figure 2: Allocation trade-off between reproduction and heat stress response in experimental evolution lines based on all significant genes.

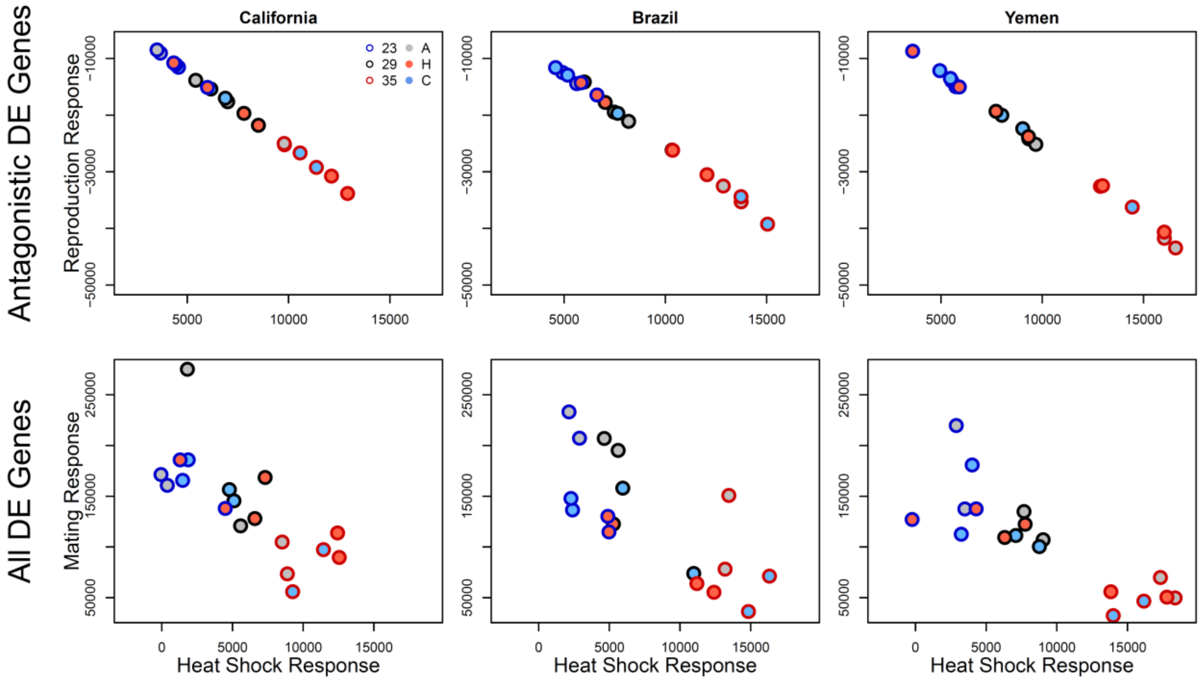

Top panels: expression responses based on the 134 antagonistic genes in hot-adapted (red fill), cold-adapted (blue fill) and ancestors (grey fill), reared at 23 (blue border), 29 (black border) or 35°C (red border). Bottom panels: the same patterns for analyses based on all genes differentially expressed in response to heat stress (765 genes) and mating (1269 genes). There was not general effect of evolution regime across the three backgrounds in either analysis, but a strong effect of rearing temperature, with allocation towards stress response and away from reproduction at 35°C. Each line's gene expression was based on transcriptomic data from a single RNA library consisting of 10 pooled female abdomens.

Supplementary Table 2: univariate models of thermal plasticity

```
############ THERMAL PLASTICITY: in Ancestral founders ###################
```

Supplementary Table 2a *### Lifetime Reproductive Success ###*

```
offspring ~ temp * origin + experiment * temp + (1 | date)
Analysis of Deviance Table (Type II Wald F tests with Kenward-Roger df)

                      F Df Df.res    Pr(>F)    
temp            33.5466  2  7.029 0.0002538 ***
origin           9.5749  2 98.301 0.0001588 ***
experiment      18.0062  2  7.672 0.0012656 ** 
temp:origin      2.5896  4 73.275 0.0435995 *  
temp:experiment 14.2318  4  8.365 0.0008606 ***
```

Supplementary Table 2b *### Development Time ###*

```
dev ~ origin * temp

              Sum Sq Df    F value    Pr(>F)    
origin          0.24  2    11.8464  0.003013 ** 
temp         1131.34  2 56919.7122 < 2.2e-16 ***
origin:temp     0.06  4     1.5213  0.275500    
Residuals       0.09  9                         
```

Supplementary Table 2c *### Metabolic Rate ###*

```
log(co2) ~ origin * temp + log(aw.mean) + (1 | Date) + (1 | Tube)
Analysis of Deviance Table (Type II Wald F tests with Kenward-Roger df)

                    F Df  Df.res    Pr(>F)    
origin         2.0253  2 102.021 0.1372207    
temp         107.8867  2   3.456 0.0007681 ***
log(aw.mean)  96.3264  1  93.599 4.745e-16 ***
origin:temp    1.7845  4  93.323 0.1385070    
```

Supplementary Table 2d *### Adult Weight ###*

```
aw.before ~ origin * temp2 + (1 | Date)
Analysis of Deviance Table (Type II Wald F tests with Kenward-Roger df)
                  F Df  Df.res    Pr(>F)    
origin       3.8808  2 103.965 0.0236895 *  
temp        28.1112  2   2.744 0.0148681 *  
origin:temp  6.2839  4 103.983 0.0001437 ***
```

Supplementary Table 2e *### Early Fecundity ###*

```
eggs ~ origin * temp2 + (1 | Tube) + (1 | Date)

Analysis of Deviance Table (Type II Wald F tests with Kenward-Roger df)
                 F Df Df.res   Pr(>F)
origin      4.7965  2 90.591 0.010471 *
temp        8.2937  2  2.949 0.061544 .
origin:temp 4.1946  4 98.993 0.003522 **
```

Supplementary Table 2f *### Water loss ###*

```
log(wvp.corr) ~ origin * temp2 + (1 | Tube)
Analysis of Deviance Table (Type II Wald F tests with Kenward-Roger df)

                 F Df Df.res Pr(>F)
origin      1.3167  2 96.187 0.2728
temp      262.1866  2 78.984 <2e-16 ***
origin:temp 1.1731  4 87.864 0.3282
```

Supplementary Table 2g *### Weigth Loss ###*

```
log(aw.loss) ~ origin * temp2 + log(aw.mean) + (1 | Tube) + (1 | Date)
Analysis of Deviance Table (Type II Wald F tests with Kenward-Roger df)

                  F Df  Df.res  Pr(>F)
origin       2.5414  2  91.487 0.08431 .
temp         8.3343  2   3.792 0.04093 *
log(aw.mean) 0.0008  1 100.813 0.97774
origin:temp  2.0975  4  98.218 0.08687 .
```

Supplementary Table 3: univariate models of thermal adaptation

```
############ ADAPTATION:  Evolution Regime Differences ###################
```

Supplementary Table 3a *### Lifetime Reproductive Success ###*

```
offspring ~ temp*origin*regime+experiment * temp + (1|pop)+(1| pop:temp)
Analysis of Deviance Table (Type II Wald F tests with Kenward-Roger df)
```

	F	Df	Df.res	Pr(>F)	
temp	22.9443	1	6.81	0.0021500	**
origin	0.7091	2	5.99	0.5291548	
regime	48.9568	1	6.23	0.0003605	***
experiment	8.7884	2	1023.46	0.0001643	***
temp:origin	2.0129	2	6.02	0.2141043	
temp:regime	1.2883	1	6.37	0.2972912	
origin:regime	0.6842	2	6.00	0.5399057	
temp:experiment	19.6974	2	1024.06	4.036e-09	***
temp:origin:regime	0.4912	2	6.02	0.6344217	

Supplementary Table 3b *### Development Time ###*

```
devtime~temp*origin*regime + (1|pop) + (1|pop:temp)
Analysis of Deviance Table (Type II Wald F tests with Kenward-Roger df)
```

	F	Df	Df.res	Pr(>F)	
temp	8926.9130	1	6	9.472e-11	***
origin	8.4124	2	6	0.01817	*
regime	10.5487	1	6	0.01751	*
temp:origin	5.0516	2	6	0.05173	.
temp:regime	108.4421	1	6	4.595e-05	***
origin:regime	0.2305	2	6	0.80084	
temp:origin:regime	2.0679	2	6	0.20743	

Supplementary Table 3c *### Metabolic Rate ###*

```
log(co2)~temp*origin*regime+log(aw)+ (1|pop)+(1|pop:temp)+(1|date)+(1|tube)
Analysis of Deviance Table (Type II Wald F tests with Kenward-Roger df)
```

	F	Df	Df.res	Pr(>F)	
temp	623.9250	1	12.55	4.435e-12	***
origin	1.0236	2	6.36	0.41175	
regime	1.8975	1	13.67	0.19050	
log(aw)	185.8547	1	328.30	< 2.2e-16	***
temp:origin	2.1438	2	6.50	0.19270	
temp:regime	1.1742	1	12.82	0.29850	
origin:regime	2.1009	2	6.54	0.19735	
temp:origin:regime	4.8510	2	7.16	0.04659	

Supplementary Table 3d *### Adult Weight ###*

```
aw ~ temp2 * origin * regime + (1 | pop) + (1 | pop:temp)
Analysis of Deviance Table (Type II Wald F tests with Kenward-Roger df)

                             F Df Df.res   Pr(>F)
temp                    4.5182  1 5.6231 0.080762 .
origin                 13.2373  2 5.9595 0.006420 **
regime                 70.9266  1 5.9626 0.000158 ***
temp:origin             7.5042  2 5.8614 0.024192 *
temp:regime            11.5186  1 5.9896 0.014644 *
origin:regime           2.4900  2 6.0374 0.162713
temp:origin:regime      3.7048  2 6.1139 0.088317 .
```

Supplementary Table 3e *### Early Fecundity ###*

```
eggs~temp*origin*regime+egg.counter+(1|pop)+(1|pop:temp)+(1|date)+(1|tube)
Analysis of Deviance Table (Type II Wald F tests with Kenward-Roger df)

                             F Df Df.res    Pr(>F)
temp                   78.7184  1  17.11 8.229e-08 ***
origin                 16.9281  2   6.07  0.003293 **
regime                 11.5846  1  17.08  0.003362 **
egg.counter            35.4965  1 343.36 6.331e-09 ***
temp:origin             0.5990  2   6.22  0.578234
temp:regime             8.2913  1  17.14  0.010344 *
origin:regime           2.6100  2   7.32  0.139386
temp:origin:regime      0.5344  2   7.37  0.607089
```

Supplementary Table 3f *### Water loss ###*

```
log(wvp)~temp*origin*regime+log(aw)+(1 |pop)+(1|pop:temp)+(1|date)+(1|tube)
Analysis of Deviance Table (Type II Wald F tests with Kenward-Roger df)

                              F Df  Df.res    Pr(>F)
temp                   941.0332  1   6.947 1.123e-08 ***
origin                   1.5900  2   5.752   0.28203
regime                  10.1036  1   8.310   0.01240 *
log(aw)                  0.9647  1 289.272   0.32684
temp:origin              0.2615  2   5.592   0.77884
temp:regime              9.3107  1   7.250   0.01777 *
origin:regime            0.3662  2   5.751   0.70845
temp:origin:regime       0.8626  2   5.827   0.46975
```

Supplementary Table 3g *### Weight Loss ###*

```
log(weight.loss+1e-
04)~temp*origin*regime+log(aw.mean)+(1|pop)+(1|pop:temp)+(1|date)+(1|tube)
Analysis of Deviance Table (Type II Wald F tests with Kenward-Roger df)

                            F Df Df.res   Pr(>F)
temp                   72.6328  1  18.35 8.47e-08 ***
origin                 10.3677  2   6.31  0.01012 *
regime                  0.9370  1  19.04  0.34519
log(aw.mean)            5.5838  1 359.36  0.01866 *
temp:origin             2.3715  2   6.26  0.17110
temp:regime             7.3232  1  17.93  0.01450 *
origin:regime           0.3279  2   6.74  0.73130
temp:origin:regime      1.1515  2   6.79  0.37102
```

Supplementary Figure 3: Alternative estimates of laboratory fitness in experimental evolution lines

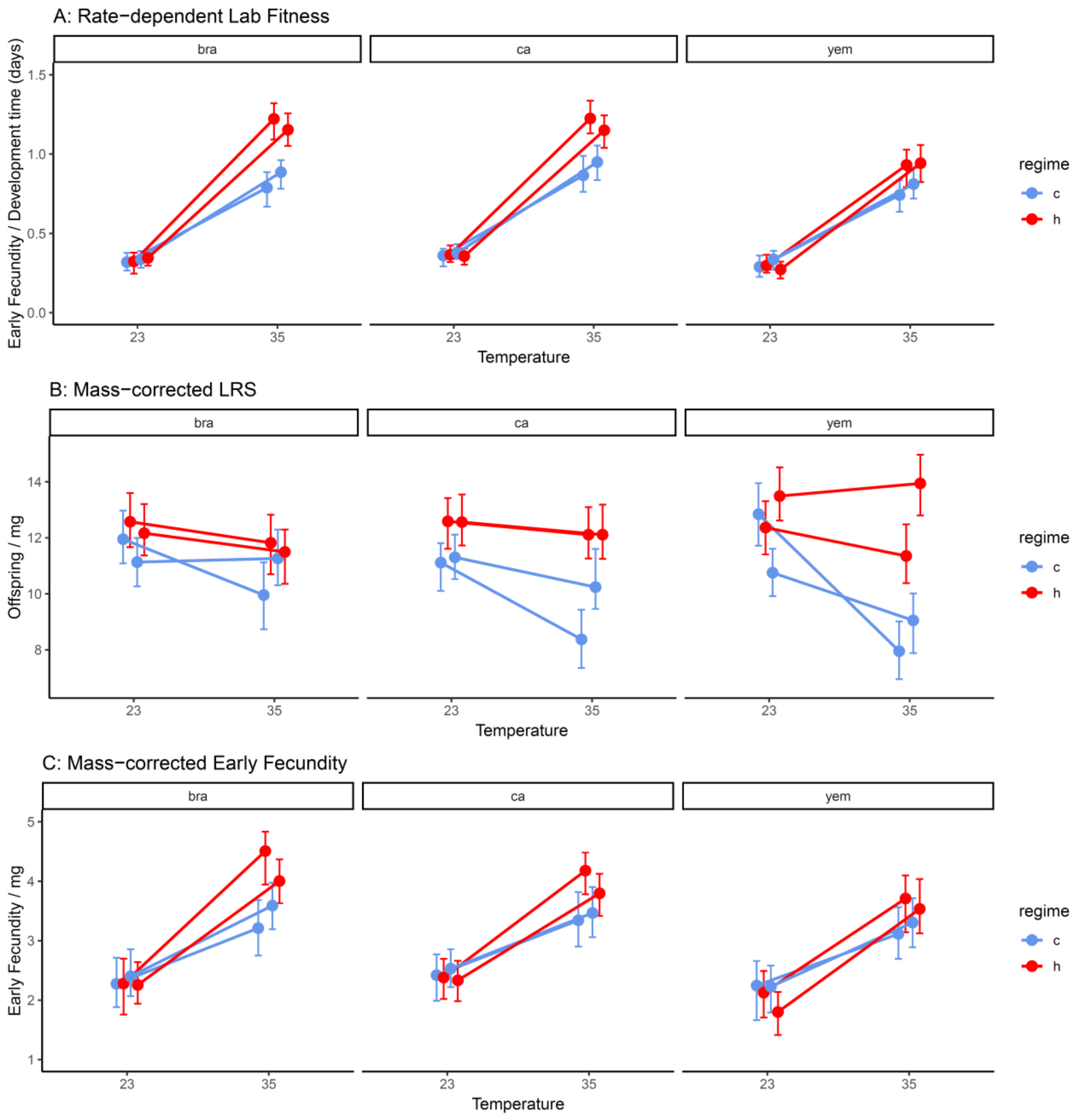

In **A)** as a proxy for fitness of each line at the conditions and two thermal conditions used in experimental evolution, we calculated a rate-dependent measure as early fecundity per female divided by development time (which corresponds roughly to generation time; mean time from start of egg laying until the emergence of reproductively mature adults) from Dataset 2. As eggs laid early should have an advantage due to being first to utilize the host resource, the calculation should give a reasonable estimate of laboratory fitness during experimental evolution. In **B**) LRS is shown corrected for mean body mass of females and in **C**) early fecundity corrected for mass is shown. All three measures show that hot-adapted lines outcompete cold-adapted lines at 35°C. Differences are less clear at 23°C, even after correcting for the larger size of females from hot-adapted lines. Whiskers represent 95% CIs based on parametric bootstrap.

Supplementary Table 4: Summary of models of host consumption in experimental evolution lines

####### Analyses of host consumption in hot and cold regime ############

Supplementary Table 4a *### Total host biomass consumed:*

```
loss.ctrl.fem ~ regime*origin*temp + (1|pop) + (1|temp:pop)
Analysis of Deviance Table (Type II Wald F tests with Kenward-Roger df)

                          F Df  Df.res    Pr(>F)   
regime              22.8112  1  5.9715 0.003115 **
origin               0.5881  2  5.9551 0.584685   
temp                 0.5359  2 11.7887 0.598759   
regime:origin        1.3612  2  5.9962 0.325530   
regime:temp          3.2465  2 11.8820 0.075016 . 
origin:temp          0.8559  4 11.8547 0.517510   
regime:origin:temp   0.4674  4 11.9324 0.758747   
```

Supplementary Table 4b *### Consumed host biomass per beetle emerging:*

```
cons.beetle ~ regime*origin*temp + (1|pop) + (1|temp2:pop)
Analysis of Deviance Table (Type II Wald F tests with Kenward-Roger df)

                          F Df  Df.res    Pr(>F)   
regime              16.3913  1  5.9410 0.006877 **
origin               0.6981  2  5.9144 0.534344   
temp                 1.9469  2 11.5953 0.186625   
regime:origin        1.4082  2  5.9818 0.315383   
regime:temp          0.5502  2 11.7669 0.590976   
origin:temp          0.6558  4 11.7140 0.634330   
regime:origin:temp   0.6467  4 11.8705 0.639931   
```

Supplementary Table 4c *### Consumed host biomass per egg laid:*

```
cons.egg ~ regime*origin*temp + (1|pop) + (1|temp:pop)
Analysis of Deviance Table (Type II Wald F tests with Kenward-Roger df)

                          F Df  Df.res     Pr(>F)    
regime              15.2788  1  5.9118  0.008137 ** 
origin               0.0864  2  5.8858  0.918403    
temp                34.6239  2 11.4288 1.412e-05 ***
regime:origin        0.4961  2  5.9513  0.632026    
regime:temp          0.0631  2 11.6719  0.939182    
origin:temp          0.9797  4 11.5938  0.455681    
regime:origin:temp   3.3172  4 11.8327  0.048229 *  
```

Supplementary Table 4d *### Consumed host biomass per beetle biomass:*

```
cons.biomass ~ regime*origin*temp + (1|pop) + (1|temp:pop)
Analysis of Deviance Table (Type II Wald F tests with Kenward-Roger df)

                          F Df  Df.res    Pr(>F)   
regime               0.5985  1  5.7284 0.469870   
origin               1.3658  2  5.6298 0.328431   
temp                10.5747  2 10.7792 0.002873 **
regime:origin        0.6121  2  5.7766 0.573975   
regime:temp          2.6759  2 11.0591 0.112765   
origin:temp          0.4299  4 10.8771 0.784332   
regime:origin:temp   1.0298  4 11.1742 0.433849   
```

####### Analyses of beetle traits in hot and cold regime ############

Supplementary Table 4e *### Minimum development time of emerging beetles:*

```
NB.DAYS.DVLP ~ regime*origin*temp + (1|pop) + (1|temp:pop)
Analysis of Deviance Table (Type II Wald F tests with Kenward-Roger df)

                               F Df Df.res     Pr(>F)
regime                    0.9963  1      6    0.35675
origin                    1.1980  2      6    0.36494
temp                    754.7321  1      6 1.538e-07 ***
regime:origin             3.5293  2      6    0.09700 .
regime:temp               1.4842  1      6    0.26885
origin:temp2              1.3395  2      6    0.33040
regime:origin:temp        3.9220  2      6    0.08141 .
```

Supplementary Table 4f *### Mean body mass of emerging beetles:*

```
beetle.size ~ regime*origin*temp + (1|pop) + (1|temp:pop)
Analysis of Deviance Table (Type II Wald F tests with Kenward-Roger df)

                               F Df Df.res     Pr(>F)
regime                   21.8705  1 5.8145 0.0036987 **
origin                    2.6164  2 5.7760 0.1552477
temp                    131.3228  1 4.7673 0.0001191 ***
regime:origin             0.5676  2 5.8428 0.5952821
regime:temp               7.8662  1 5.0982 0.0369797 *
origin:temp              12.4183  2 4.7663 0.0128767 *
regime:origin:temp        1.1448  2 5.1029 0.3884123
```

Supplementary Table 4g *### Model explaining host consumption from life history traits:*

#fecundity ="eggs"" #Mean adult mass ="beetle.size" #juvenile survival = "survival"

```
loss.ctrl ~ scale(beetle.size)+scale(survival)+scale(eggs)+temp

                   Estimate Std. Error t value Pr(>|t|)
(Intercept)         0.97480    0.01037  94.027  < 2e-16 ***
scale(beetle.size)  0.07982    0.01121   7.117 2.08e-10 ***
scale(survival)     0.10423    0.01117   9.331 4.45e-15 ***
scale(eggs)         0.41430    0.01309  31.641  < 2e-16 ***

Residual standard error: 0.1031 on 95 degrees of freedom
  (10 observations deleted due to missingness)
Multiple R-squared:  0.9273,	Adjusted R-squared:  0.925
F-statistic: 403.7 on 3 and 95 DF,  p-value: < 2.2e-16
```

Supplementary Figure 4: Mean body mass of beetles emerging from the assays of host consumption.

|

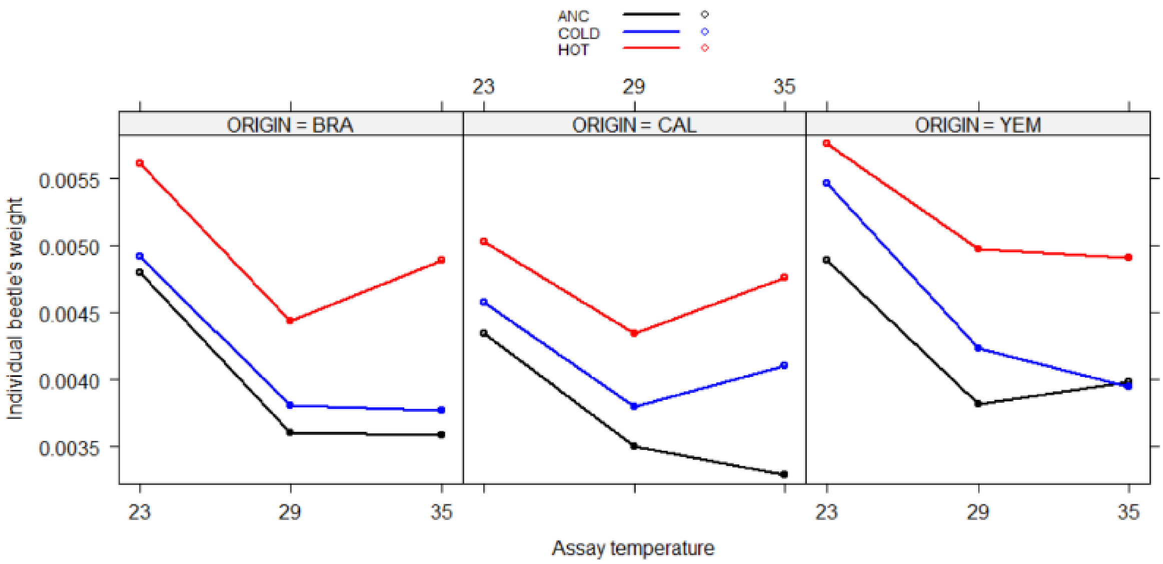

Shown a means for each regime on each genetic background. Note that the ancestors (black) are included for illustrative purposes but were not included in the statistical analyses of cold (blue) and hot (red) regimes, presented in the anova tables

Supplementary Figure 5: Selection on female body mass at different temperatures

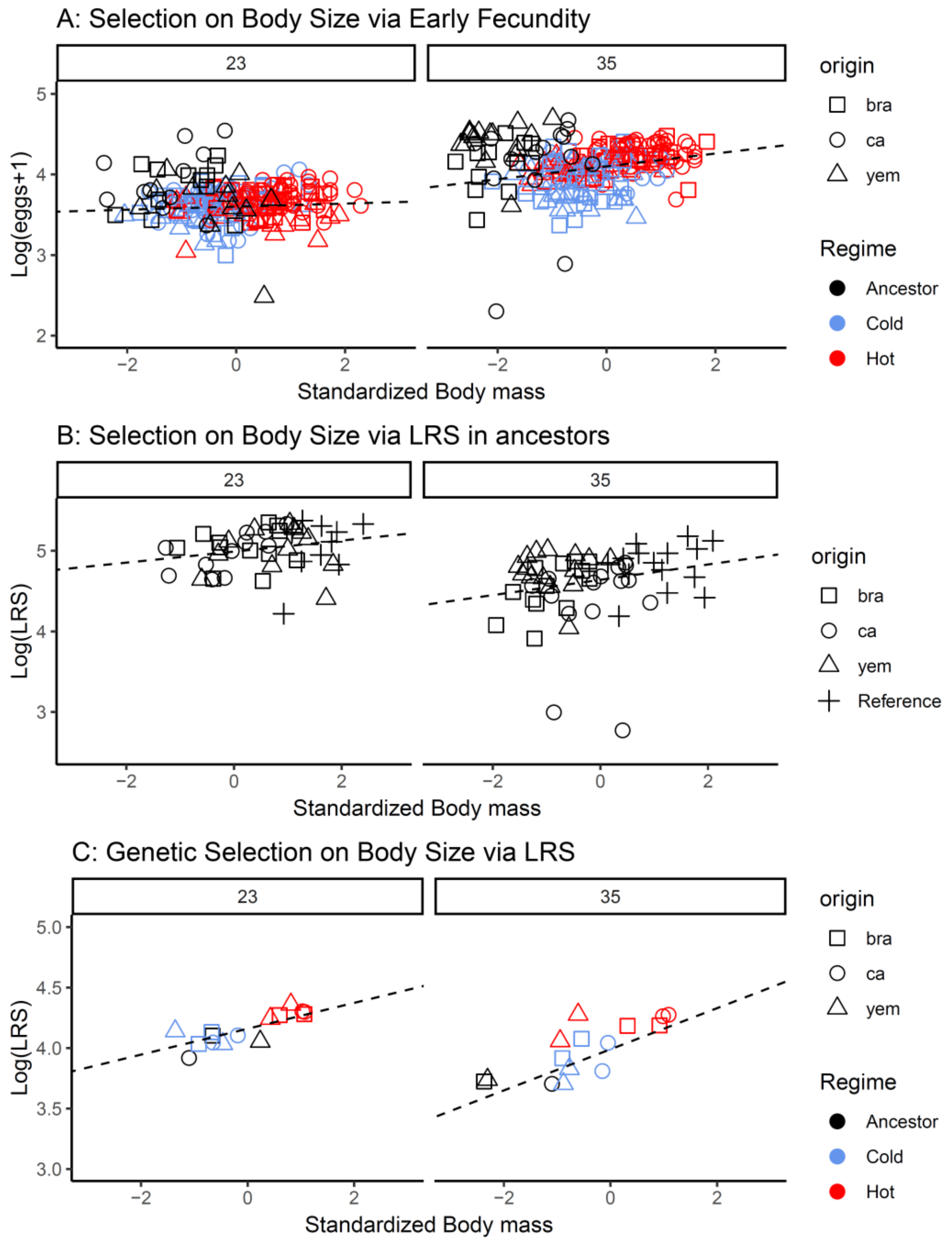

A) Regression of logarithmized early fecundity on variance standardized body mass for each female trio from dataset 1 & 2. **B**) Regression of logarithmized lifetime reproductive success (number of adult offspring produced) and variance standardized body mass for female trios from the experiment on ancestral populations that measured both traits on the same individuals. **C**) Regression of logarithmized mean lifetime reproductive success on mean (variance standardized) body mass for each line used in the experiment. Selection gradients on size are given by the regression slopes. These slopes were estimated across all data. In panel A, this was done after first removing effects of evolution regime (cold/hot/ancestor). In each case the selection gradient is steeper at 35°C compared to 23°C.

Supplementary Table 5: Selection on adult body mass

Supplementary Table 5a

```
### Selection on body mass (aw) via early fecundity in evolution lines ###
### Corresponds to panel A of Figure S6 ###

log(eggs + 1) ~ temp*scale(aw)+selection*temp

                         Estimate Std. Error t value Pr(>|t|)
(Intercept - ancestor)   3.82066    0.04461  85.636  < 2e-16 ***
temp35                   0.47389    0.06821   6.948 1.29e-11 ***
scale(aw)                0.02080    0.02303   0.903  0.36694
regime.c                -0.20117    0.04993  -4.029 6.56e-05 ***
regime.h                -0.18576    0.05943  -3.126  0.00189 **
temp35:scale(aw)         0.05512    0.03186   1.730  0.08432 .
temp35:regime.c         -0.13462    0.07497  -1.796  0.07322 .
temp35:regime.h          0.04767    0.08700   0.548  0.58399

Residual standard error: 0.2475 on 456 degrees of freedom
Multiple R-squared:  0.496,     Adjusted R-squared:  0.4882
F-statistic:  64.1 on 7 and 456 DF,  p-value: < 2.2e-16


Anova Table (Type II tests)
                 Sum Sq  Df  F value     Pr(>F)
temp            21.7727   1 355.4800 < 2.2e-16 ***
scale(aw)        0.5945   1   9.7067  0.001952 **
regime           3.6228   2  29.5747  8.38e-13 ***
temp:scale(aw)   0.1833   1   2.9927  0.084316 .
temp:regime      0.7136   2   5.8252  0.003176 **
Residuals       27.9294 456
```

Supplementary Table 5b

```
#at 23C:

            Estimate Std. Error t value Pr(>|t|)
(Intercept)  3.82377    0.04125  92.707  < 2e-16 ***
scale(aw)    0.01923    0.01912   1.006 0.315681
reimge.c    -0.20117    0.04484  -4.486 1.15e-05 ***
regime.h    -0.18576    0.05337  -3.480 0.000601 ***

Residual standard error: 0.2223 on 226 degrees of freedom
Multiple R-squared:  0.08268,   Adjusted R-squared:  0.0705
F-statistic:  6.79 on 3 and 226 DF,  p-value: 0.0002109
```

Supplementary Table 5c

```
#at 35C:

            Estimate Std. Error t value Pr(>|t|)
(Intercept)  4.28339    0.05393  79.420  < 2e-16 ***
scale(aw)    0.07973    0.02523   3.160  0.00179 **
regime.c    -0.33578    0.06101  -5.504  9.9e-08 ***
regime.h    -0.13808    0.06932  -1.992  0.04755 *

Residual standard error: 0.27 on 230 degrees of freedom
Multiple R-squared:  0.1872,    Adjusted R-squared:  0.1765
F-statistic: 17.65 on 3 and 230 DF,  p-value: 2.396e-10
```

Supplementary Table 5d

```
### Selection on body mass (aw) via LRS in ancestors ###
### Corresponds to panel B of Figure S6 ###


log(total.fecundity+1) ~ temp *scale(aw.before)

                         Estimate Std. Error t value Pr(>|t|)    
(Intercept)               4.98807    0.06024  82.809  < 2e-16 ***
temp35                   -0.34478    0.07855  -4.389 2.85e-05 ***
scale(aw.before)          0.06846    0.06275   1.091    0.278    
temp35:scale(aw.before)   0.02664    0.07984   0.334    0.739    

Residual standard error: 0.3645 on 99 degrees of freedom
Multiple R-squared:  0.2666,	Adjusted R-squared:  0.2443 
F-statistic: 11.99 on 3 and 99 DF,  p-value: 9.177e-07


Anova Table (Type II tests)

Response: log(total.fecundity + 1)
                        Sum Sq Df F value    Pr(>F)    
temp                    2.5541  1 19.2210 2.905e-05 ***
scale(aw.before)        0.6367  1  4.7919   0.03094 *  
temp:scale(aw.before)   0.0148  1  0.1114   0.73928    
Residuals              13.1551 99                      
```

Supplementary Table 5e

```
#at 23C:

                 Estimate Std. Error t value Pr(>|t|)    
(Intercept)       5.01642    0.03976 126.179   <2e-16 ***
scale(aw.before)  0.05996    0.04021   1.491    0.143    

Residual standard error: 0.2667 on 43 degrees of freedom
Multiple R-squared:  0.04917,	Adjusted R-squared:  0.02706 
F-statistic: 2.224 on 1 and 43 DF,  p-value: 0.1432
```

Supplementary Table 5f

```
#at 35C:

                 Estimate Std. Error t value Pr(>|t|)    
(Intercept)       4.61273    0.05575  82.733   <2e-16 ***
scale(aw.before)  0.09304    0.05624   1.654    0.104    

Residual standard error: 0.4246 on 56 degrees of freedom
Multiple R-squared:  0.04659,	Adjusted R-squared:  0.02956 
F-statistic: 2.736 on 1 and 56 DF,  p-value: 0.1037
```

Supplementary Table 5g

```
### Genetic selection on body mass (aw) via LRS ###
### Corresponds to panel C of Figure S6 ###


log(LRS) ~ temp*scale(aw)

                    Estimate Std. Error t value Pr(>|t|)
(Intercept)          4.12760    0.03152 130.946  < 2e-16 ***
temp35              -0.10609    0.04432  -2.394  0.02418 *
scale(aw)            0.12201    0.03590   3.398  0.00219 **
temp235:scale(aw)    0.03371    0.04608   0.731  0.47107

Residual standard error: 0.118 on 26 degrees of freedom
Multiple R-squared:  0.6828,     Adjusted R-squared:  0.6462
F-statistic: 18.65 on 3 and 26 DF,  p-value: 1.158e-06


Anova Table (Type II tests)

Response: log(LRS)
                 Sum Sq Df F value    Pr(>F)
temp            0.07752  1   5.570   0.02605 *
scale(aw)       0.55746  1  40.057 1.059e-06 ***
temp:scale(aw)  0.00744  1   0.535   0.47107
Residuals       0.36183 26
```

Supplementary Table 5h

```
#At 23C:

            Estimate Std. Error t value Pr(>|t|)
(Intercept)  4.15518    0.01961  211.93  < 2e-16 ***
scale(aw)    0.10715    0.02029    5.28 0.000149 ***

Residual standard error: 0.07593 on 13 degrees of freedom
Multiple R-squared:  0.682,      Adjusted R-squared:  0.6575
F-statistic: 27.88 on 1 and 13 DF,  p-value: 0.000149
```

Supplementary Table 5i

```
#At 35C:

            Estimate Std. Error t value Pr(>|t|)
(Intercept)  3.98632    0.03836  103.93  < 2e-16 ***
scale(aw)    0.16991    0.03970    4.28 0.000896 ***

Residual standard error: 0.1485 on 13 degrees of freedom
Multiple R-squared:  0.5849,     Adjusted R-squared:  0.5529
F-statistic: 18.32 on 1 and 13 DF,  p-value: 0.0008964
```
