# Supplementary material for: Life-history adaptation under climate warming magnifies the agricultural footprint of a cosmopolitan insect pest
Source: Nat Commun. 2025 Jan 18;16:827. doi: 10.1038/s41467-025-56177-2 (PMC11743133; doi:10.1038/s41467-025-56177-2)
Supplement: Supplementary file 2 — Description of Additional Supplementary Files [file 41467_2025_56177_MOESM2_ESM.pdf]

Description of Additional Supplementary Files

Supplementary Data 1: Gene annotations

Gene annotations for all heat stress and reproduction genes.

Supplementary Data 2: gene ontology with FDR

Gene ontology for all heat stress and reproduction genes.

Supplementary Code 1: Theoretical predictions of thermal adaptation and agricultural impact

R-code for producing model predictind of agricultural impact presented in Figure 1 of main text and Supplementary Note 1.
