# Supplementary material for: Life-history adaptation under climate warming magnifies the agricultural footprint of a cosmopolitan insect pest
Source: Nat Commun. 2025 Jan 18;16:827. doi: 10.1038/s41467-025-56177-2 (PMC11743133; doi:10.1038/s41467-025-56177-2)
Supplement: Supplementary file 6 — Reporting Summary [file 41467_2025_56177_MOESM6_ESM.pdf]

nature portfolio

Corresponding author(s): David Berger
Last updated by author(s): Nov 20, 2024

Reporting Summary

Nature Portfolio wishes to improve the reproducibility of the work that we publish. This form provides structure for consistency and transparency in reporting. For further information on Nature Portfolio policies, see our Editorial Policies and the Editorial Policy Checklist.

Statistics

For all statistical analyses, confirm that the following items are present in the figure legend, table legend, main text, or Methods section.

n/a	Confirmed	
☐	☒	The exact sample size (*n*) for each experimental group/condition, given as a discrete number and unit of measurement
☐	☒	A statement on whether measurements were taken from distinct samples or whether the same sample was measured repeatedly
☐	☒	The statistical test(s) used AND whether they are one- or two-sided<br>*Only common tests should be described solely by name; describe more complex techniques in the Methods section.*
☐	☒	A description of all covariates tested
☐	☒	A description of any assumptions or corrections, such as tests of normality and adjustment for multiple comparisons
☐	☒	A full description of the statistical parameters including central tendency (e.g. means) or other basic estimates (e.g. regression coefficient) AND variation (e.g. standard deviation) or associated estimates of uncertainty (e.g. confidence intervals)
☐	☒	For null hypothesis testing, the test statistic (e.g. *F*, *t*, *r*) with confidence intervals, effect sizes, degrees of freedom and *P* value noted<br>*Give P values as exact values whenever suitable.*
☒	☐	For Bayesian analysis, information on the choice of priors and Markov chain Monte Carlo settings
☐	☒	For hierarchical and complex designs, identification of the appropriate level for tests and full reporting of outcomes
☐	☒	Estimates of effect sizes (e.g. Cohen's *d*, Pearson's *r*), indicating how they were calculated

Our web collection on statistics for biologists contains articles on many of the points above.

Software and code

Policy information about availability of computer code

Data collection	R and Rstudio (v. 2022.07.2 Build 576). Open source.<br>Code for model predictions available as Supplementary Information. See "Code availability" statement in manuscript.
Data analysis	R and Rstudio (v. 2022.07.2 Build 576). Open source.<br>Code available at Figshare. https://doi.org/10.6084/m9.figshare.26048389

For manuscripts utilizing custom algorithms or software that are central to the research but not yet described in published literature, software must be made available to editors and reviewers. We strongly encourage code deposition in a community repository (e.g. GitHub). See the Nature Portfolio guidelines for submitting code & software for further information.

Data

Policy information about availability of data

All manuscripts must include a data availability statement. This statement should provide the following information, where applicable:

- Accession codes, unique identifiers, or web links for publicly available datasets
- A description of any restrictions on data availability
- For clinical datasets or third party data, please ensure that the statement adheres to our policy

Accession codes to all data have been provided - see "Data availability" statement in manuscript.

Research involving human participants, their data, or biological material

Policy information about studies with human participants or human data. See also policy information about sex, gender (identity/presentation), and sexual orientation and race, ethnicity and racism.

Reporting on sex and gender	NA
Reporting on race, ethnicity, or other socially relevant groupings	NA
Population characteristics	NA
Recruitment	NA
Ethics oversight	NA

Note that full information on the approval of the study protocol must also be provided in the manuscript.

Field-specific reporting

Please select the one below that is the best fit for your research. If you are not sure, read the appropriate sections before making your selection.

☐ Life sciences ☐ Behavioural & social sciences ☒ Ecological, evolutionary & environmental sciences

For a reference copy of the document with all sections, see nature.com/documents/nr-reporting-summary-flat.pdf

Ecological, evolutionary & environmental sciences study design

All studies must disclose on these points even when the disclosure is negative.

Study description

12 Experimental evolution lines and their 3 ancestal lines, of the seed beetle Callosobruchus maculatus, were analyzed.
6 lines were evovled at hot (35C) temperature, and 6 lines were evolved at cold (23C) temperature. The ancestors were kept at ancestral lab temperautre(29C). The evovled lines were created from the ancestors, that originally were sampled from Brazil, Yemen and California (USA). Thus experimental evolution was carried out on three different genetic backgrounds, with two biological replicates per evolution regime and and background.

Evolution lines were scored for their life history adaptation by measuring traits at generations 45-60 and again for lifetime offspring production at generations 80-120. Ancestors were measured for their traits at generation 120.

We scored how life history traits impact host plant consumption for all lines in a common garden at generations 80-120. (the number of generations differ depending on the line assayed, but the actual time of data collection was the same for all lines).

We modelled how life history adaptation affects allocation and aqcuisition trade-offs and how this in turn affects host plant consumption rate.

To test the model-assumption of a temperature dependent trade-off between investment on reproduction and maintenance, we used geen expression data from three independent datasets. Two datasets were from a population sampled from Lome, Togo. The third dataset was from the current evolution lines and their ancestors.

Research sample

Callosobruchus macualtus seed beetles (as desribed above). 12 evolved + 3 ancestral lines. Most measurements were done on females, as these are more directly affecting population growth rates. C. macualtus is a cosmopolitan pest on legume crops. We therefore compared how evolution affects its agricultural footprint across three genetic backgrounds sampled from Brazil, Yemen and California.

Two datasets on gene expression come from a fourth additional population, sampled from Lome, Togo. Gene expression was measured on female abdomens throughout.

Sampling strategy

We measured several thousands of beetles to attain relatively accurate estimates of means for all 7 life-history traits of each line. For these traits, our lab has measured them before, we therefore had a good idea of what was needed, even though no direct power analysis was conducted. Having that said, we also were limited practically, so we also pushed on and tried to measure life history traits for as many beetles as possible for the assays of metabolic rate and related traits.

Similarly, for estimating host plant consumption, we again set up all lines as the focus was to represent all lines in the analysis (lines represent the true level of replciation in experimental evolution experiments), and then we simply tried our best to replicate each line to the best of our ability. For life history traits, we focused on representing as many adult females as possible.

For measuring host plant consumption, we instead tried to focus on measuring the effect of as many larvae as possible, so the number of mothers of these larvae was much smaller. Thus, as stated in the manuscript, adult fecundity/offspring production, was

much better estiamted in the first experiments on life history trait evolution, comapred to the last experiment on host plant consumption.

Gene expression data was limited by costs foremost. Hence, we tried to maximize statistical power by pooling individuals into RNA libraries and aimed at havinh biological replicates for each line in each temperature at minimum. We note that power was high for answering the main question (finding the trade-off).

Data collection

Gene expression
To provide evidence for a temperature-dependent trade-off between reproduction and maintenance, we leveraged transcriptomic data from three separate experiments. The first two experiments estimated gene expression responses in female abdomens to heat shock and mating, respectively, in a separate lab population collected from Lomé, Togo. The third dataset estimated expression in female abdomens in response to rearing temperature in our experimental evolution lines.

To identify heat stress responsive genes, we compared reproductively active control and heat shocked females. Individuals were collected as virgins and kept individually. On day 2, females were heat shocked in individual 60 mm petri dishes at 55° C for 20 minutes, which is stressful and reduces female fertility101,102. After a 2.5 h at 29° C and 55% rh, all females were mated to males. Females were then kept in individual 60 mm dishes until being flash frozen in liquid nitrogen 2-3 h, 6-7 h or 24-25 h after mating. The experiment was carried out in three consecutive blocks. Each replicate sample consisted of 5 pooled female abdomens from the same treatment, time-point and day, resulting in a total of seven replicate samples per time-point for the control treatment and five replicate samples per time-point for the heat shock treatment. Preliminary analyses showed that there were only three differentially expressed genes at the last time-point, which therefore was removed in the final analyses (although results did not qualitatively change if including them), resulting in 24 RNA libraries.

To identify reproductive genes, we compared virgin and mated females 24 h after a single mating. We mated females to males from 8 different genetic lines. The lines had undergone 53 generations of experimental evolution under one of three mating regimes manipulating the relative strength of natural and sexual selection. Accordingly, we had 8 samples of mated females, with 2 or 3 samples per male mating regime. Additionally, we had 3 samples of virgin control females. Flash freezing of all females occurred 24 h after mating, with females kept singly and allowed to lay eggs on fresh beans during the 24h period. The final 11 RNA libraries each consisted of 12 female abdomens.

To quantify expression of reproductive and heat stress responsive genes (identified in the first two datasets) in our experimental evolution lines and their ancestors, we reared all lines at 23, 29 and 35°C. All lines were first propagated for one generation at 29°C to remove potential difference between evolution regimes stemming from temperature-induced (non-genetic) parental effects. Once adults emerged at respective rearing temperature, 5 males and 5 females were placed together in a 90mm petri dish with access to fresh beans. Beetles were allowed to reproduce for 23h (for 29 and 35°C) or 46h (for 23°C) after which females were flash frozen. The longer time allowed for reproduction at cold assay temperature was given to make a more direct comparison across females from different temperatures; reproductive output, metabolism and weight loss is about twice as fast at 35°C compared to 23°C. Two petri dishes of females were pooled into one sample (i.e. 10 female abdomens pooled in total) for extractions and further analysis, resulting in one sample per line and temperature for the Hot and Cold regime. Two samples were taken per temperature for each of the three ancestors. This resulted in a total of 54 RNA libraries.

RNA from all samples was extracted using the Qiagen RNeasy Mini kit with beta-mercapto-Ethanol added to the lysis buffer and an on-column DNase treatment with the Qiagen RNase-free DNase kit. Tissue lysis was done in a bead mill with two stainless steel beads at 28 Hz for 90 s and RNA was eluted in two times 30-50 µl water. Some samples went through an additional clean-up step with the Qiagen RNeasy Mini Kit after the RNA extraction. Next generation sequencing of the samples was done at the SNP&SEQ Technology Platform in Uppsala. Libraries were prepared using the TruSeq stranded mRNA library preparation kit with polyA selection. Libraries were sequenced in one flowcell as paired-end 150 bp reads on a NovaSeq 6000 system.

Raw reads were quality checked with FastQC 0.11.9 and MultiQC 1.11 and afterwards cleaned up using Trimmomatic 0.39. Adapters were trimmed from the raw reads and ends below an average accuracy of 99% over a five base pair sliding window were clipped. Finally, reads shorter than 20 bp were discarded. Resulting read pairs were mapped to the Callosobruchus maculatus genome using HISAT2 2.2.1 with default settings for stranded libraries and sensitivity set to "--very-sensitive". Number of reads per gene was then determined using HTSeq 2.0.2 with default settings for stranded libraries. This resulted in 10-27M (heat stress), 13-30M (reproduction) and 9-35M (rearing temperature during experimental evolution) uniquely mapped reads per library going into the final downstream analyses.

Read count data was pre-processed and analysed in R 4.3.1 using edgeR. For the heat shock response, only genes with at least 3 counts per million in at least 5 samples were further analysed, resulting in 10246 genes for final analysis. For the mating response, only genes with at least 1 count per million in at least 2 samples were further analysed, resulting in 11640 genes for final analysis. The last approach was also used for the genes expressed in the evolution regimes, resulting in 11292 genes for analysis. Counts were normalized using the 'Trimmed Mean of M-values' method via edgeR's NormLibSizes() function. Count data for the heat shock and mating response was further analysed with edgeR and limma using linear models on the normalized log2 transformed counts per million to determine differentially expressed genes. All analyses used a false discovery cut-off of 5 %.

For reproductive genes, effect sizes (i.e. Log-fold changes) were averaged across the three male selection regimes. However, we only considered genes that were significantly differentially expressed in all three separate contrasts between virgin females and females mated to males from the three mating regimes. For heat stress genes, effect sizes and significance was calculated by averaging across the two time-points. Significant genes were further subject to gene ontology analysis, using the R package GOstats (2.66.0) and the HyperGTest function. Results were visualised using clusterProfiler (4.10).

To provide a powerful, yet, unbiased estimate of temperature-dependent allocation between reproduction (mating response) and maintenance (heat stress response) in the experimental evolution lines, we focused on the 134 genes that displayed significant antagonistic differential expression patterns in response to heat shock and mating. We first multiplied the estimated log fold changes of the antagonistic genes for both mating and heat shock responses by the vector of normalized read counts of the same 134

antagonistic genes found for each of the 54 RNA libraries from the experimental evolution lines. The resulting values were then summed to compute a score for the heat stress and reproduction response separately. As expected, the scores for heat stress and reproduction, based on only the antagonistic genes, were tightly negatively correlated (r = -0.999) across all combinations of evolved lines and assay temperatures and fell along a singular linear trade-off axis. The response along the allocation trade-off for each sample was taken by projecting samples along this axis (quantified as the first eigenvector of the data). We repeated the same analyses using all differentially expressed genes in response to heat shock (765) and mating (1269) with qualitatively similar results.

Life-history traits
We quantified thermal adaptation in female life-history by measuring three core traits: lifetime reproductive success (LRS), juvenile development time and adult body mass, and four rate-dependent traits: early fecundity, weight loss, water loss, and mass-specific metabolic rate (ml CO_2/mass/min) over the first 16h of female reproduction. All life-history traits were collected at generations 40 for cold-adapted lines and 60 for hot-adapted lines, in a large common garden experiment including the two assay temperatures corresponding to the experimental evolution treatments (23°C and 35°C). Ancestral lines were scored in the same experimental conditions with the addition of the ancestral 29°C assay temperature, but on a later occasion following ca. 125 generations of experimental evolution. Note that the ancestors had been kept at the ancestral conditions, to which they had already adapted for more than 300 generations prior to the start of experimental evolution. It can therefore be assumed that the measured trait values correspond well with the trait values at the start of experimental evolution. To control for potential differences in the separate experiments on ancestors and evolved lines stemming from unknown sources, we reared an independent laboratory adapted reference population in both experiments. This indicated that differences in rearing had affected the life-history traits scored over the first 16h of reproduction. We therefore standardized the traits scored during respirometry of the three founding ancestors by this estimated amount (adult mass: increased by 6.4%, metabolic rate: reduced by 12%; early fecundity: reduced by 18%; water loss: reduced by 15%, and weight loss: reduced by 25%) in order not to erroneously assign these differences to evolutionary divergence between ancestors and evolved lines. Note that this was done averaged across the three assay temperatures and geographic origins. Therefore, our approach to provide more accurate measures of evolutionary divergence between evolved lines and ancestors did not affect the estimated temperature-dependence of adaptation or the importance of geographic differences.

Before assays of life-history traits, non-genetic parental effects were removed by moving F0 grandparents of the assayed individuals into a common temperature of 29°C to lay eggs. The emerging beetles in the next (parental) F1 generation were allowed to mate and lay eggs on beans provided ad libitum. Following 48 hours of egg laying, the beans were split and assigned to one of the two (for ancestors, three) assay temperatures. The emerging adult F2 offspring were phenotyped for their life-history. Newly emerged (0-48 hours old) virgin females were mated to males by placing three males and females together in a petri dish over night at the assay temperature. In the following morning, the three females were weighed for their body mass and then placed together inside a glass vial filled with black eyed beans to be measured for their metabolic rate, water loss and early fecundity at their designated assay temperature. The glass vials were placed in a Sable Systems (Las Vegas, NV, USA) high-throughput respirometry system. Briefly, the respirometry was set up in stop-flow mode, and CO_2 production and water-loss was measured for up to 23 vials on a given experimental day. The first vial was left empty and served as a baseline to control for any drift of the gas analysers during each session. Vials were measured over 17 cycles, each of a length of 57.5 minutes. Mean metabolic rate and water loss for each vial was calculated across cycles 2-17, with the readings from the first cycle discarded (as it contains human-produced water and CO_2). After respirometry, females were weighed again to record their weight loss and beans with eggs were isolated and counted to record early fecundity. In total we followed 386 triads of females for the evolved lines and another 115 triads from their ancestors.

From the same rearing we measured egg-to-adult development time for two technical replicates per line and assay temperature, each consisting of 40-120 individuals. We calculated a mean development time per technical replicate and used this in analysis. We also collected virgin males and females and placed three males and three females together in a petri dish with ad libitum beans to record lifetime reproductive output (LRS) at each assay temperature. In total we recorded LRS for 258 couple triplets for evolved lines, and another 115 couple triplets for the ancestors. These data were complemented with additional data from both evolved and ancestral lines reared in a common garden design in two consecutive years (corresponding to generation 120/135 for ancestors, 115/130 for hot-adapted lines, and 80/90 for cold-adapted lines). In these rearings, a single male and female were put together in a petri dish with ad libitum host seeds, with otherwise the same conditions. For ancestors we scored 396 couples, and for evolved lines 789 couples, across both experimental years. LRS was analysed per female, hence we divided all offspring counts from female triads by three before analysis.

We carried out all statistical analyses using the statistical and programming software R (v. 3.6.1). All seven traits were first analysed separately using linear mixed effect models available in the lme4 package115. When analysing differences between hot- and cold-adapted lines, evolution regime, geographic origin and assay temperature were added as fully crossed fixed effects. Line replicate crossed with assay temperature and nested within geographic origin were added as random terms to assure correct level of replication when estimating significance of fixed terms including evolution regime. For analyses on metabolic rate, water loss and weight loss, the date of the respirometry run was added as an additional random effect. Metabolic rate and weight loss were corrected for body mass of the measured female triad by taking an average of the weight measures before and after respirometry and adding it as a covariate in analysis. For water loss measured from evolved lines, measures from vial 2 (the first vial in the measuring sequence containing beetles) and measurements from three entire experimental days were discarded following an outlier analysis as these measures were magnitudes greater than other measures and clearly represented water vapour from other sources than the beetles. For LRS, experiment (triads and two separate rearings of single couples) and its interaction with assay temperature were added as additional fixed effects to account for possible block effects. The data from ancestral lines were analyzed in similar fashion but without the fixed effect of evolution regime and random effect of population replicate.

P-values were calculated using the car package and type II sums of squares with the Kenward-Roger approximation for the degrees of freedom. To analyse evolutionary responses while taking all traits into consideration at once, we also performed a non-parametric MANOVA using residual randomization with the same structure for fixed and random effects.

The agricultural footprint
We set up both evolved and ancestral lines in a common garden experiment including the 23, 29 and 35°C assay temperature, following approximately 85, 120 and 125 generations of experimental evolution for cold-adapted, hot-adapted and ancestral lines, respectively. We removed parental effects by moving all lines to 29°C two generations prior to the start of the experiment. Newly laid

F2 eggs were split among the three assay temperatures and resulting virgin adults were collected. Three males and three females were placed together in petri-dishes with ad libitum V. unguiculata seeds for 24h at 29°C and 35°C, and 34h at 23°C (as reproductive rate is slower at cold temperature; Fig. 4B). Three dishes were prepared per line and assay temperature. Note that the experiment was aimed at linking life-history variation to host consumption, and the sample size was low for detecting more fine-scaled differences between evolution regimes due to the imprecise measures of fecundity (6-9 females per line and temperature measured over 24-34h), which were much better estimated in the previous experiments (~80 females per line and temperature across the entire adult stage).

To quantify the amount of host seed consumed by beetles, each petri dish with beans was weighed prior to the addition of beetles, to record a starting weight of each assay. After beetles had emerged and been removed, assays were placed in standard conditions for 6 weeks during which they were weighed on several occasions to record the weight of the infested beans and to check for potential inconsistencies and time-dependencies in estimates of bean consumption. However, the measures of the beans' weight loss following infestation were highly repeatable (r2 = 94%) and we chose the final (fourth) measure to calculate our estimate of host consumption. To account for potential weight loss of beans that was independent of the beetle infestation, we also set up 5 assays without beetles at each temperature and measured them in the exact same way. The host consumption was then corrected based on the weight changes in these control assays; this correction was very small and only corresponded to 1-2% of the total bean weight. For each assay, we also recorded the number of hatched eggs laid on beans (i.e. fecundity), the number of those that resulted in the adult pupating (i.e. juvenile survival), and the mean body mass of emerged adult beetles.

Differences in food consumption were first analyzed using linear mixed effect models in the lme4 package with the same structure for fixed and random terms as described for life-history traits. We excluded the ancestors from formal analysis since these had only half the samples compared to the evolution regimes. We sequentially added information on fecundity, juvenile survival, and mean body mass for each assay to explore how life-history traits accounted for host consumption. We finally fitted a linear model including only the three traits (removing the fixed terms of assay temperature, evolution regime and geographic background) to estimate how much of the variation in host consumption that could be predicted by life-history alone.

Motivated by the observation that the life-history traits accounted for more than 93% of the observed variation in host consumption, we calculated the two independent measures of the agricultural footprint (see main text) from our measured life-history traits in the main experiment, as this data was estimated with high accuracy for all lines. To provide 95% confidence limits and P-values, we performed parametric bootstrap on all calculations using the means and standard errors for each trait per line and assay temperature derived from the univariate mixed models.

Timing and spatial scale	Life history traits were scored in April-June 2018 (generations 45-60) for evovled lines, and for ancestors in April-May 2022. Because ancestors and evovled lines were not scored at the same time for these traits, we also included data for lifetime time offspring produciton measured in a common garden (including both) at generation January-March 2023. During this time, we also collected the gene expression data on all lines + ancestors (raised at 23, 29 and 35C, common garden).<br><br>Host plant consumption was assayed in in all lines and ancestors in a common garden conducted in Sept 2021- Jan 2022.<br><br>Gene expression data on reproductive genes from the Lome population: completed July 2020, sequenced Nov. 2020.<br>Gene expression data on heat-stress genes from the Lome population: completed July 2021, sequenced Jan 2022.
Data exclusions	We excluded single (repeated measures) data from certain runs of the metabilic rate equipment on "water-loss", since these were several magnitude off. This is likely due to human-created moisture (e.g. breatng into the system set-up when loading samples) in the measuring tubes that sit first in sequence. This is described in the manuscript.
Reproducibility	As described above, we measured lifetime offspring production twice to verify that differences remained betwen evolution regimes and ancestors. We also used a "standard genetic line" in both experiments on evolved lines (G45-60) and ancestors (G120) to remove experimental/environmental effects (as described in manuscript). We also compared differences between lines for traits that were measutred in both the experiment on life history traits and host consumption; the qualitative differences between evolution regimes and ancestors were very similar.
Randomization	We reared populations and always tried to run samples from each line on the same day. This was not possible to do on all daya as the lines have evolved differences in development time, and beetles develop at differnet rates in different temperatures. Hence, while lines were started at the same time in the experiment, they were not finishing at the same time, and for some traits they were measured on different days.
Blinding	For all assays we used ID numbers and not the actual name of the lines. However, since beetles develop predictably from differnet temperature treatments, it was not possible to blind the observer from this particular aspect of the experimental design.

Did the study involve field work? ☐ Yes ☒ No

Reporting for specific materials, systems and methods

We require information from authors about some types of materials, experimental systems and methods used in many studies. Here, indicate whether each material, system or method listed is relevant to your study. If you are not sure if a list item applies to your research, read the appropriate section before selecting a response.

Materials & experimental systems

n/a	Involved in the study
☒	☐ Antibodies
☒	☐ Eukaryotic cell lines
☒	☐ Palaeontology and archaeology
☐	☒ Animals and other organisms
☒	☐ Clinical data
☒	☐ Dual use research of concern
☒	☐ Plants

Methods

n/a	Involved in the study
☒	☐ ChIP-seq
☒	☐ Flow cytometry
☒	☐ MRI-based neuroimaging

Animals and other research organisms

Policy information about studies involving animals; ARRIVE guidelines recommended for reporting animal research, and Sex and Gender in Research

Laboratory animals	Callosobruchus maculatus (strains from Brazil, Yemen, USA and Togo).
Wild animals	did not involve
Reporting on sex	Only females were measured except for development times, and the effects on host plant consumption which were averaged across both sexes.
Field-collected samples	Laboratory stocks which have been maintained >10 years before experiments (according to standard lab conditions - 29C and 50-55% RH)
Ethics oversight	No ethical approval is necessary for insects according to national legislation.

Note that full information on the approval of the study protocol must also be provided in the manuscript.

Plants

Seed stocks	*Report on the source of all seed stocks or other plant material used. If applicable, state the seed stock centre and catalogue number. If plant specimens were collected from the field, describe the collection location, date and sampling procedures.*
Novel plant genotypes	*Describe the methods by which all novel plant genotypes were produced. This includes those generated by transgenic approaches, gene editing, chemical/radiation-based mutagenesis and hybridization. For transgenic lines, describe the transformation method, the number of independent lines analyzed and the generation upon which experiments were performed. For gene-edited lines, describe the editor used, the endogenous sequence targeted for editing, the targeting guide RNA sequence (if applicable) and how the editor was applied.*
Authentication	*Describe any authentication procedures for each seed stock used or novel genotype generated. Describe any experiments used to assess the effect of a mutation and, where applicable, how potential secondary effects (e.g. second site T-DNA insertions, mosiacism, off-target gene editing) were examined.*
